# A machine learning approach to graduate admissions and the role of letters of recommendation

**DOI:** 10.1371/journal.pone.0291107

**Published:** 2023-10-25

**Authors:** Yijun Zhao, Xiaoyu Chen, Haoran Xue, Gary M. Weiss

**Affiliations:** Computer and Information Sciences Department, Fordham University, New York, NY, United States of America; Bahria University - Lahore Campus, PAKISTAN

## Abstract

The graduate admissions process is time-consuming, subjective, and complicated by the need to combine information from diverse data sources. Letters of recommendation (LORs) are particularly difficult to evaluate and it is unclear how much impact they have on admissions decisions. This study addresses these concerns by building machine learning models to predict admissions decisions for two STEM graduate programs, with a focus on examining the contribution of LORs in the decision-making process. We train our predictive models leveraging information extracted from structured application forms (e.g., undergraduate GPA, standardized test scores, etc.), applicants’ resumes, and LORs. A particular challenge in our study is the different modalities of application data (i.e., text vs. structured forms). To address this issue, we converted the textual LORs into features using a commercial natural language processing product and a manual rating process that we developed. By analyzing the predictive performance of the models using different subsets of features, we show that LORs alone provide only modest, but useful, predictive signals to admission decisions; the best model for predicting admissions decisions utilized both LOR and non-LOR data and achieved 89% accuracy. Our experiments demonstrate promising results in the utility of automated systems for assisting with graduate admission decisions. The findings confirm the value of LORs and the effectiveness of our feature engineering methods from LOR text. This study also assesses the significance of individual features using the SHAP method, thereby providing insight into key factors affecting graduate admission decisions.

## Introduction

The graduate admissions process is frequently time-consuming and subjective. Furthermore, the lack of standard scoring systems makes it challenging to properly weigh the various application materials and compare applicants evaluated by different committee members. An application typically consists of structured form data such as prior grades, undergraduate major, standardized test scores, and textual data such as a resume, statement of intent, and letters of recommendation (LORs). LORs are a key component of graduate program admissions but can be particularly difficult to evaluate due to the varying roles of the recommender (e.g., instructor versus employer) and their knowledge of the applicant, and the subjectivity involved in interpreting the recommendations.

In this study, we apply machine learning techniques to application data to assist the admissions process of two graduate programs offered by Fordham’s Computer and Information Sciences department: a Master’s in Computer Science (MSCS) and a Master’s in Data Science (MSDS). The admissions process we currently follow, which we consider representative of many modest-sized Master’s programs, includes a dedicated team of full-time faculty members from the Department of Computer and Information Sciences. Most applications undergo two reviews, and the decision-making process involves a holistic evaluation of the application materials without predetermined scoring or strict cutoff values for individual components. While a minimum undergraduate GPA of 3.0 is generally expected, exceptions can be made based on factors such as relevant work experience, research experience, and the reputation of the applicant’s undergraduate institution. Each reviewer examines all application materials, including standardized test scores, statements of intent, resumes, letters of recommendation, and prior transcripts. Reviewers provide concise rationales for their decisions, and any disagreements are resolved through further discussion or by the program director after considering the provided rationales.

This study aims to develop automated machine learning models for predicting admission decisions based on application materials used in the current process. There are two primary challenges in utilizing application data for building these models. Firstly, the data consists of multiple modalities (i.e., structured and textual), necessitating effective fusion to ensure accurate model performance. Secondly, the subjective nature of interpreting LORs and the lack of use of standardized guidelines [1] add complexity to the process. To address these challenges, we adopted quantitative measures by leveraging IBM’s natural language processing (NLP) techniques to extract features from the LORs. Additionally, we established guidelines for rating each LOR based on relevance, specificity, and positivity, promoting consistent scoring. Keyword matching software was used to record if specific keywords were present in the resumes. These extracted features from textual data were then integrated with structured data elements from the electronic application. Machine learning algorithms were then applied to the combined features and existing admissions decisions to build predictive models. The acquired model can be used for future data to generate admissions decisions for new applications. The machine learning algorithms effectively learn how to best combine the various application components, although this generally does not involve learning explicit weights as in a regression analysis. This systematic approach provides consistency in the decision-making process and thus can reduce some of the subjectivity associated with the admission committee’s evaluations. It also provides insight into the relative importance of the various application components in making the admissions decision.

Another key motivation for this study is to assess the impact of LORs on graduate admission decisions. We place a special focus on LORs because, unlike quantifiable items such as GPAs or standardized test scores, LORs are the most heterogeneous and subjective components of an admissions application. As a result, their role in the decision-making process is hard to assess or characterize. Our study introduces an innovative approach by employing advanced NLP techniques to characterize LORs. While there has been limited prior research on quantifying the influence of LORs on admission decisions, as discussed in the subsequent section on related work, the utilization of automated NLP technology remains largely unexplored. Hence, a substantial part of our research is devoted to obtaining, integrating, and assessing the influence of LOR features on admission decisions. Our evaluation involves analyzing predictive models using the LORs alone, the non-LOR components alone, and the combined dataset. Our findings suggest that LORs in isolation provide only modest predictive signals compared to the non-LOR components, but nonetheless provide a boost in predictive performance when combined with the non-LOR data.

Lastly, we analyze the contribution of individual features to the model output using the SHAP method, thereby providing insight into key factors when the models decide to accept or reject an applicant. While the approach described in this study can be deployed to render admissions decisions, we advocate a more conservative approach, discussed in the conclusion section, where machine learning is used to guide and assist with the admissions decisions.

## Related work

Machine learning has been used in prior work to predict admissions decisions for graduate programs [2]; undergraduate programs [3]; MBA programs [4]; and computer science undergraduate [5] and graduate [6, 7] programs. One of these studies of graduate computer science admissions was quite extensive and considered 150, 000 computer science graduate admissions applications spanning 3000 institutions [7]. However, the diversity of the applications prevented a direct analysis of the application components and instead the study relied on self-reported outcomes for a few components such as test scores and undergraduate grades, while textual data sources like LORs or resumes were not incorporated.

A distinctive aspect of our study is the use of textual admissions components, especially the LORs. There is some prior work that also considers the LORs in the context of making admissions decisions. We begin by describing those studies that utilize manual rating systems to characterize the LORs. One study of graduate students in various programs at the University of California at Los Angeles assigned a manually determined numerical score to each LOR and found that the LOR was the least significant factor of the seven features that were employed in the predictive model [2]. A study of thoracic surgery residency programs had program directors manually rate the importance of various admissions criteria and the study found that applicant interview performance, letters of recommendation, and professionalism were found to be very important [8]. A small study of twenty-four orthopaedic residency graduate programs had reviewers manually rate each LOR as “strong” or “exceptional” based on guidelines that they developed and found that applicants with three or more strong letters of recommendation had slightly higher admission scores [9].

One large-scale study of LORs performed a meta-analysis of previously published research spanning undergraduate and graduate education [10]. Unlike our study, the goal was not to use LORs as predictors of past admission decisions, but rather as predictors of future performance. The study found that LORs have low, but positive, correlations with standardized test scores and moderate (i.e., 0.26) correlations with prior grades. LORs also had low but positive correlations with future performance, including a correlation of 0.10 with research productivity, 0.28 with undergraduate GPA, 0.13 with graduate GPA, and 0.19 with completion of a doctoral degree. An analysis of the incremental validity of LORs over the other predictors like test scores and past GPA showed that the LORs do not substantially help with predicting future graduate GPA but do help with predicting graduate degree completion. As degree completion is one of our key goals when making admissions decisions, this is quite notable.

None of the prior studies described thus far utilized NLP techniques to characterize the LORs, indicating a significant gap in research in this field. Waters and Miikkulainen presented an admission-decision study, where they applied NLP techniques to LORs using a statistical machine learning approach to facilitate large-scale Ph.D. admissions [6]. Their system incorporated numerical, categorical, and textual data, with the LOR text transformed into a 50-dimensional feature vector using a bag-of-words representation (i.e., word order is not considered) and Latent Semantic Analysis [11] techniques. The study found that LORs containing words such as “best,” “award,” “research,” “PhD,” etc., were predictive of admission, while letters containing words like “good,” “class” “programming,” “technology,” etc., were indicative of rejection. According to the authors, this pattern reflects the faculty’s preference for candidates with strong research potential. The use of NLP in this study was relatively straightforward as the focus was on specific words.

Several studies utilized more advanced NLP techniques on LORs, but these studies were specifically in the context of investigating gender and racial bias in LORs. These bias-related studies used NLP software to assess the linguistic characteristics of the LORs, including those related to emotional content (e.g., sadness, excitement). The majority of these studies focused on graduate medical programs [12–15], while a few studies considered graduate STEM disciplines [16, 17] and one focused on undergraduate admissions [18]. The study on undergraduate admissions [18] for the University of California at Berkeley showed that LORs written for students in underrepresented racial groups were weaker than those for other students. However, this study also assessed the impact of LORs on admission decisions and showed that even though the LORs were weaker for these underrepresented groups, the inclusion of LORs nonetheless improved the admission outcomes for these students.

The influence of resumes on admissions decisions has received less attention than the impact of LORs, potentially because not all programs mandate a resume submission. One such study manually annotated resume data to monitor one specific skill, research experience [2]. In contrast, our study examines nine skills extracted automatically from the resume text.

## Data and feature extraction

This study is based on 717 applications submitted to the MSCS and MSDS programs of Fordham University, a major metropolitan university located in New York City. The gender breakdown is 37% female and 63% male. The applicants rarely specified the optional “Race” field of the application, resulting in a lack of precise racial breakdown. To account for missing race values, we introduced a new racial category, “unknown,” which is then used in our models. Applicants with a permanent residence in the United States make up 63% of the applicants and have been observed to be racially diverse, which may be related to the university being located in a racially diverse city. The models and performance results in this study are based on this one dataset, and different programs would require retraining of the models.

The application data has three main components: application form data, resume, and letters of recommendation. Each of these components is described in successive subsections, along with the features and, if necessary, the mechanism for extracting the features. Table 1 presents the complete list of features employed in this study.

**Table 1:** Features used by the machine learning model.

Application Form and Resume Data (Non-LOR Data)			LOR Data		
**Demographics**	**GRE Scores**	**Resume Skills**^1^ **(CS)**	**Manual Ratings**	**NLP Tones** ^2^	**Other**
Gender	Quantitative %	Python	Positivity	t-Excited	Length
Race	Verbal %	Java	Relevance	t-Frustrated	
Age	Analytical %	C++	Specificity	t-Impolite	
Permanent Country		Matlab		t-Polite	
Native English Speaker	**Resume Skills**^1^ **(Math)**	SAS	**NLP Emotions** ^2^	t-Sad	
	Calculus	Database	e-Anger	t-Satisfied	
**Academic Credentials**	Linear Algebra	Microsoft Office	e-Disgust	t-Sympathetic	
Undergraduate Major	Statistics	Machine Learning	e-Fear		
Undergraduate GPA		Software	e-Joy	**NLP** ^2^	
Months Since Degree			e-Sadness	Sentiment	

^1^ Each skill represents a binary feature indicating if the applicant’s resume contains the keyword.

^2^ Analysis performed using IBM NLU Tookit [19]. “e-” and “t-” indicate emotion and tone variables, respectively.

### Application form data and features

The application form data is already well structured, so no feature extraction is required. The features are logically partitioned into three categories, as shown in Table 1. The “demographic” features include an applicant’s gender, race, age, permanent country, and a binary feature that indicates whether they are a native English speaker. The “academic credentials” features include the undergraduate major, GPA, and the number of months since completing the last degree, which will typically be an undergraduate degree but could be a graduate degree. The “GRE Scores” include the three graduate record exam (GRE) components: the quantitative, verbal, and analytical scores, each expressed as a percentile.

### Resume data and features

Each applicant is required to submit a resume, which is a pdf file containing text and hence is considered to be unstructured, or more precisely, semi-structured data. Most machine learning classification algorithms cannot directly utilize unstructured information, so features are extracted using a simple pattern matching program that looks for specific keywords. The list of keyword features is provided in Table 1 and is partitioned into Math and Computer Science (CS) skills. The keywords were selected because they appear in many resumes and are technically relevant to the degree programs. These keyword features are binary and indicate the presence or absence of the keyword in the applicant’s resume.

### Letter of recommendation data and features

Each application typically has two or three recommendations, which are semi-structured text. Thirteen features are automatically generated using a commercial NLP product from IBM [19] and three features are extracted via a labor-intensive manual rating process. Finally, the length of the LOR is estimated using a character counting program.

#### Automatically generated LOR features

We applied IBM Watson’s Natural Language Understanding (NLU) service [19] to extract linguistic characteristics from the LORs. Specifically, NLU utilizes deep learning models [20] (i.e., neural networks with many layers) to provide tone, emotion, and sentiment scores for features listed in Table 1. The tone and emotion scores are assigned real values between 0 and 1. The tracked emotions are anger, disgust, fear, joy, and sadness. The seven tones are not as self-explanatory as the emotions, and we reproduce their definitions from the NLU tone analytics document here:

- *Excited*—showing personal enthusiasm and interest
- *Frustrated*—feeling annoyed and irritable
- *Impolite*—being disrespectful and rude
- *Polite*—displaying rational, goal-oriented behavior
- *Sad*—an unpleasant passive emotion
- *Satisfied*—an affective response to perceived service quality
- *Sympathetic*—an affective mode of understanding that involves emotional resonance

The final feature extracted by the NLU software is the sentiment score, a numerical value representing the overall emotional undertone expressed in the LOR. The score ranges from -1 (most negative) to 0 (neutral) and up to +1 (most positive).

We utilized the default NLU model (version 2022-04-07) to extract the Emotion and Sentiment features and the English tone analytics model (tone-classifications-en-v1) to obtain the scores of the seven tones. However, the tone model is limited to only analyzing the first 2000 text characters. To overcome this limitation, we divided long LORs into smaller components and utilized NLTK’s sentence tokenizer to ensure the splits occurred at sentence boundaries. The final score for each letter is determined by the average of all component scores, with each score weighted by the length of its respective component.

#### Manually rated LOR features

We designed three categorical features, *positivity*, *relevance*, and *specificity*, to describe each letter of recommendation. Positivity measures the overall positive sentiment of the recommendation and a key indicator of its value often appears near the end of the LOR text when the recommender summarizes their recommendation. Since both our experience and prior work [10] shows that the majority of LORs are positive, our positivity scale focuses on varying levels of positive sentiment. Relevance indicates how relevant the LOR is to the specific program being applied to and the skills and knowledge necessary to succeed in the program. Since the Master’s degree programs in this study are highly technical, relevant technical skills are given priority over personal qualities such as being a hard worker, and LORs from instructors of relevant coursework are generally considered more relevant than academic letters associated with other coursework or employer letters from non-technical positions. Specificity measures the extent to which the LOR contains specific and detailed information.

These features are independently rated using nominal values based on guidelines developed by our research group. These guidelines included sample LORs that specified the expected relevance, specificity, and positivity ratings, accompanied by explanations for the assigned values. Table 2 summarizes the possible ratings of each feature and provides a brief description for each value. To minimize inter-rater variability, the raters participated in practice sessions where they rated LORs and then discussed any differences to align their interpretations of the guidelines and calibrate their ratings. The final ratings were generated by a team of nine research assistants over a period of two months. Due to the substantial effort involved, each LOR was rated by only a single person. In practice, the best recommendation letter will have excellent relevance, excellent specificity, and very strong positivity, whereas an LOR with excellent specificity and very strong positivity, but minimal relevance, would be of very little value.

**Table 2:** Definition of manually rated LOR features.

Value	Description
Positivity	
Weak	Negative or weakly positive; Trying to put a positive spin.
Positive	Several positives but below average; “I recommend the student.”
Strong	Above average positivity; “I enthusiastically recommend the student.”
Very Strong	Unusually positive, exceptional; “Student is in top 5% I have taught.”
Relevance	
Minimal	Little relevant info for making decision; Focuses on non-technical skills.
Good	Some relevant info for making decision; Covers technical skills.
Excellent	Extremely helpful for making decision. Covers variety of technical skills
Specificity	
Poor	Form letter with almost no specifics;
Average	Few specifics, relies mainly on grades, appears to barely no student.
Good	Several specific statements; Modest knowledge of student as individual.
Excellent	Many specifics; Clearly knows applicant well.

## Methods

This section describes the methods used in the study. We first introduce the machine learning algorithms and the methodology used to apply them. Next, we describe the evaluation metrics and the SHAP plots used to assess the role of each feature in the models.

This study was approved by Fordham’s Institutional Review Board, and informed consent was waived. All procedures were carried out following relevant guidelines and regulations. To comply with the Family Educational Rights and Privacy Act (FERPA), we anonymized student and recommender identities in the data by automatically redacting their names and removing affiliations.

### Machine learning model and techniques

We investigated different machine learning models for our classification task, including logistic regression [21], Naive Bayes [22], Decision Tree [23], Random Forest [24] and XGBoost [25]. All experiments were conducted using 10-fold cross-validation and we report the average model performance across the test folds. Bagging [26] was used to address the data imbalance issue between the two classes [27]. Specifically, 100 balanced bags were created, and each bag consisted of all minority class instances and an equal number of randomly sampled majority class instances. Subsequently, 100 submodels were trained on each balanced bag, and the final prediction was rendered using majority voting on the output of the submodels. The target variable utilized for our classification task is the admission outcome associated with each application.

It is typical for each application to carry multiple recommendation letters. Thus, when we train the model using LORs, the model outputs decisions at the LOR level, while the admission decisions must be rendered at the application level. To address this discrepancy, an ensemble algorithm is used to generate the application-level prediction from the LOR-level predictions. We explored two methods to combine the individual learners’ predictions for application-level decisions. The first method employed a combination of majority voting and average predictive probability. Specifically, if there were an odd number of LORs associated with an application, majority voting was used; otherwise, the decision was made based on the higher average predictive probability of the two classes. The second method utilized the average predictive probability in all scenarios, regardless of the number of LORs associated with an application. Our findings indicated that the former method yielded superior results, so that method was used to generate the results presented in the Results section. One possible explanation for this outcome is that the majority voting approach assigns equal weight to each LOR, whereas the average probability method can be influenced by an overly positive or negative letter.

The input data to the combined model consist of the complete list of features extracted from the non-LOR (i.e., application-level) components and LORs. This part of the study suffers the same issue as in the LORs-only model because each applicant may submit multiple LORs. To address this, we individually joined the application-level features with each set of *k* LOR features associated with the application to generate *k* training instances from each application. These *k* instances shared the same decision label (i.e., accept or reject). The complete list of input features is shown in Table 1. We applied the same ensemble method described above to aggregate the model predictions from the LOR level to the application level.

### Model evaluation metrics

The efficacy of our predictive models are evaluated using the following five metrics applied to the test data. The “admit” and “reject” decisions correspond to the positive and negative classes, respectively.

- *Accuracy*: measures the proportion of total instances that are correctly predicted by a model. It provides a general measure of how well the model performs across all classes or categories.
- *Recall*: measures the proportion of true positive instances that are correctly identified by a classification model. A high recall indicates that the model is effectively identifying a large portion of positive instances from the dataset.
- *Specificity*: measures the proportion of true negative instances that are correctly identified by a classification model. A high specificity indicates that the model is effectively identifying a large portion of negative instances from the dataset.
- *PPV* (Positive Predictive Value): also know as *precision*, measures the proportion of true positive predictions (correctly identified positive instances) out of the total predicted positive instances. It indicates how likely a positive prediction is to be accurate.
- *NPV* (Negative Predictive Value): measures the proportion of true negative predictions (correctly identified negative instances) out of the total predicted negative instances. It indicates how likely a negative prediction is to be accurate.
- *F1 score*: assesses the accuracy of a model by considering both *precision* and *recall*. It offers a balanced evaluation by taking into account the harmonic mean of these two metrics.

### Model interpretation with SHAP plots

**Fig 1:**
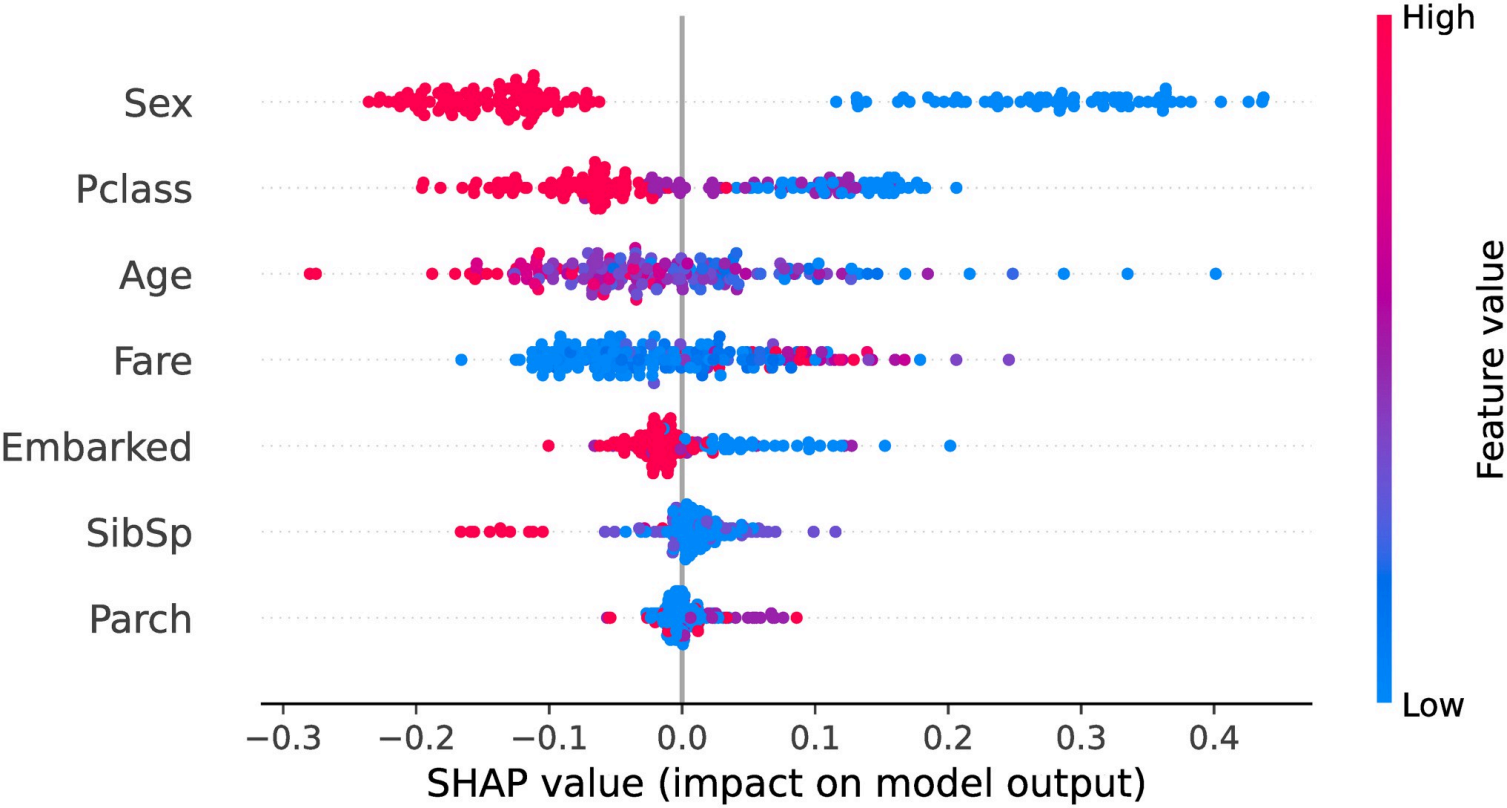
SHAP plot for Titanic survival prediction.

SHAP (SHapley Additive exPlanation) is a framework to interpret the predictions of machine learning models [28], and is used in our study to identify the key features for making admissions decisions. In SHAP analysis, a model is a coalition game in which accurate prediction is the goal and the predictive features are the players. The SHAP plot effectively visualizes the individual player’s contributions to the game’s outcomes. For example, Fig 1 illustrates the beeswarm SHAP plot for a random forest model applied to predicting a passenger’s survival status in the tragic Titanic accident. The dependent variables are 12 characteristic features (Sex, Pclass, Age, etc.) of each passenger. Fig 1 plots the seven most predictive features in their order of relative importance along the y-axis. Each row illustrates a feature’s contribution to the predictive outcomes in which each dot represents an instance in the dataset. Feature values are color-coded from blue (low) to red (high). We observe that a passenger’s sex (encoded as male = 0 and female = 1) is the most predictive feature in predicting a passenger’s chances of survival. Low values (i.e., female) are concentrated on the right side of the y-axis, indicating a high probability of survival. The same is true for passengers with low age and low Pclass (encoded as first-, second-, and third-class cabin) values. Thus, we can infer that women, children, and passengers in first-class cabins had a greater chance of survival than the others.

## Results

This section provides our main results. We first compare the efficacy of predictive models using three subsets of the application data, which permits us to assess the overall impact of the LOR data. We next study the contribution of individual features on the admissions decisions by analysing the principal predictors.

### Model performance

Table 3 presents the results of the predictive models trained using the following three sets of features:

- Non-LOR: contains features extracted from structured application forms and the semi-structured resume data. We use this model as a baseline performance benchmark.
- LORs: contains only features extracted from the LOR data. The purpose of this model is to confirm the presence of predictive signals in LORs for admissions decisions.
- Combined: contains all features associated with non-LOR and LOR data. By comparing the models using the combined features with those using only non-LOR features, we can determine the incremental predictive value of the LORs.

**Table 3:** Performance of machine learning models in predicting admissions decisions.

	Non-LOR	LORs	Combined	Gain**
	(NL)	(L)	(C)	(C over NL)
	Random Forest			
Accuracy	0.83	0.60	**0.89**	**7.2%**
Recall*	0.84	0.61	**0.90**	**7.1%**
Specificity*	0.71	0.56	**0.82**	**15.5%**
PPV*	0.96	0.91	**0.97**	**1.0%**
NPV*	0.38	0.16	**0.52**	**36.8%**
F1*	0.90	0.73	**0.93**	**3.3%**
	Naive Bayes			
Accuracy	0.67	0.54	**0.68**	**1.5%**
Recall	0.68	0.53	**0.68**	0.0%
Specificity	0.61	0.61	**0.67**	**9.8%**
PPV	0.93	0.91	**0.94**	**1.1%**
NPV	0.20	0.15	**0.22**	**10.0%**
F1	0.78	0.67	**0.79**	**1.3%**
	Logistic Regression			
Accuracy	0.77	0.56	**0.80**	**3.9%**
Recall	0.78	0.56	**0.80**	**1.3%**
Specificity	0.68	0.57	**0.79**	**16.2%**
PPV	0.95	0.91	**0.97**	**2.1%**
NPV	0.29	0.15	**0.34**	**17.2%**
F1	0.86	0.69	**0.87**	**1.2%**
	Decision Trees			
Accuracy	0.80	0.62	**0.87**	**8.8%**
Recall	0.81	0.63	**0.87**	**7.4%**
Specificity	0.73	0.57	**0.86**	**17.8%**
PPV	0.96	0.92	**0.98**	**2.1%**
NPV	0.33	0.17	**0.47**	**42.4%**
F1	0.88	0.75	**0.92**	**4.6%**
	XGBoost			
Accuracy	0.80	0.62	**0.87**	**8.8%**
Recall	0.81	0.63	**0.87**	**7.4%**
Specificity	0.75	0.61	**0.85**	**13.3%**
PPV	0.96	0.92	**0.98**	**2.1%**
NPV	0.34	0.18	**0.46**	**35.3%**
F1	0.88	0.75	**0.92**	**4.6%**

*Positive class = *“admit”*

**Calculated using (C-NL)/NL

Table 3 shows that there is a consistent gain when using the combined data over the other two types of data, across all evaluation metrics and machine learning models. Given the similarities in patterns, our analysis focuses on the results produced by the random forest model, which exhibited relatively high overall accuracy and precision compared to other models. Specifically, the model achieved an 83% overall accuracy when employing non-LOR data, 60% when considering only the LOR data, and 89% when utilizing the combined data. Thus, the addition of the LOR data yields a 6% absolute increase and a 7.2% relative increase in predictive accuracy. Thus, even though the LOR data has a relatively weak predictive signal, it nonetheless provides a meaningful improvement in overall accuracy when combined with the other data. This can only occur if the information contained in the LOR data is different, and largely independent of, the information in the non-LOR data, as ensemble classifiers benefit most when the information sources are diverse. This outcome is not particularly surprising, given that well-written LORs provide a view of the applicant that could not be obtained from the structured data. For example, an LOR could explain why an applicant performed poorly in one semester due to external circumstances, which might have a large impact on the admissions decision. Finally, the relative gains calculated for the combined data are limited for some metrics due to their close-to-optimal baseline benchmarks. For instance, the increase of F1 from 0.90 to 0.93 is a 3.3% relative improvement, but brings the score 30% closer to the optimal value of 1.0.

The model’s performance for the admitted (i.e., recall) and rejected (i.e., specificity) classes follow the same pattern as the overall accuracy. Using combined data, the gain over non-LOR data for the admitted class is 7.1%. The corresponding improvement for the rejected class (i.e., specificity) is 15.5%. All three models exhibit high precision (i.e., PPV) scores with 0.96, 0.91, and 0.97 for the non-LOR data, LORs, and combined data, respectively. Compared to PPV, the overall lower NPV scores result from the negative class being the minority class. Nevertheless, we observe a significant 36.8% improvement after incorporating LORs into the structured data.

### Principal predictor analysis

We are interested in studying the principal predictors for our machine learning models because they provide insight into the key factors affecting admissions decisions. To this end, we utilize the SHAP method to rank features that made an above-average contribution to the random forest model’s output and examine their directional impact on the decision-making process. Table 4 presents the list of such features in each model. We present the top 10 (out of 18) principal predictors for the combined model.

**Table 4:** Above-average contributors to model output.

Non-LOR	LORs	Combined (top 10)
Undergrad GPA	Sentiment	Undergrad GPA
GRE Verbal %	Length	GRE Quant %
GRE Quant %	t-Sad	GRE Verbal %
GRE Analytical %	t-Impolite	Age
Java		Java
Age		GRE Analytical %
Matlab		Relevance
Time Since Degree		Matlab
		e-Fear
		Time Since Degree

#### Using non-LOR data

The first column in Table 4 shows eight (out of 23) above-average contributing features when using structured data. Of these, academic performance (i.e., undergraduate GPA) and standard test results (i.e., GRE percentiles) are the most significant predictors. The other four features suggest the importance of students’ programming skills (i.e., Java and Matlab) and their continued student status (i.e., Age and Time Since Degree).

**Fig 2:**
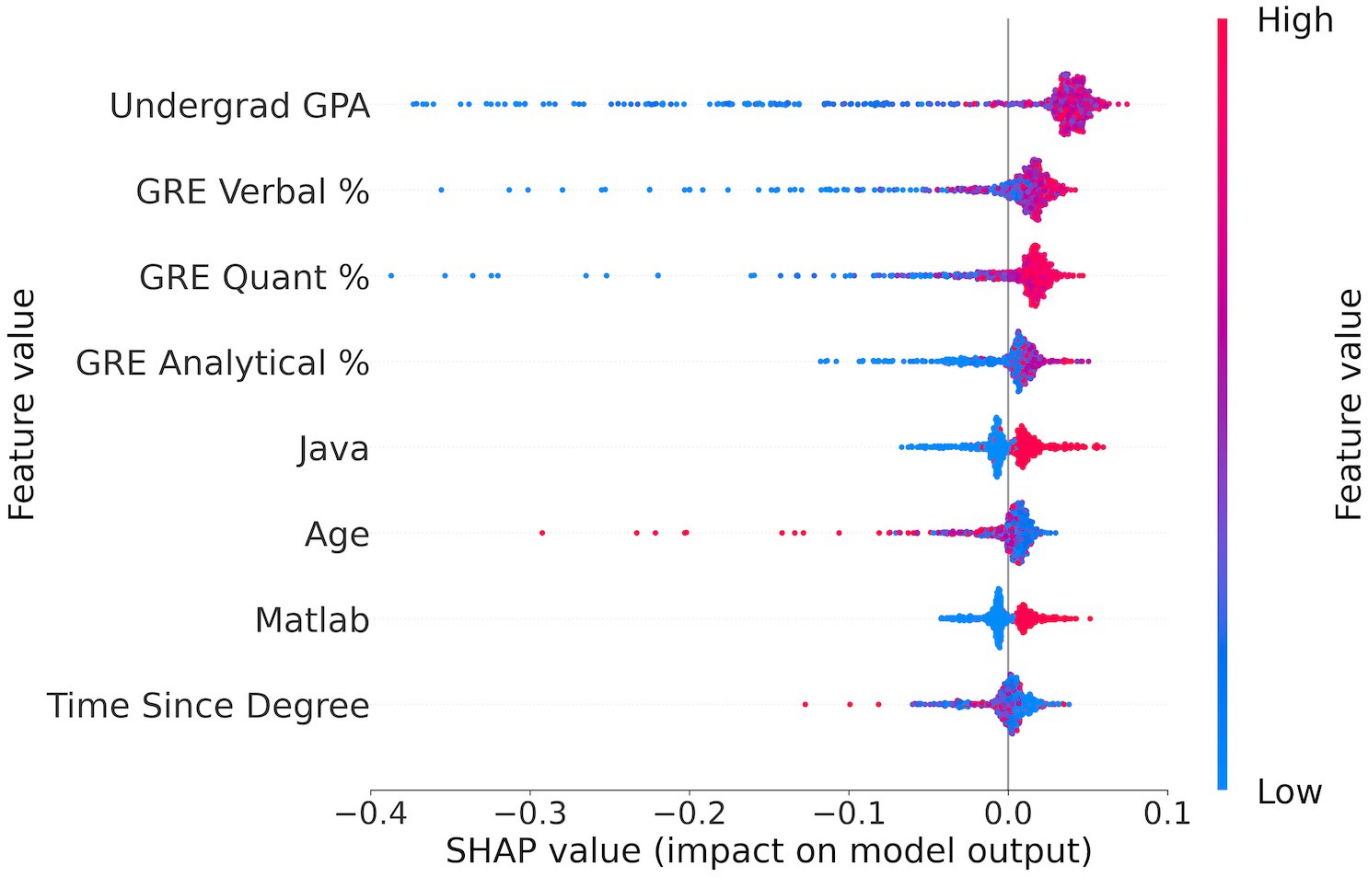
Principal predictors for admissions prediction using non-LOR data.

Fig 2 reveals six features are positive predictors because their high feature values (red) are concentrated on the right side of the y-axis. The importance ranking of these positive features, from highest to lowest, is: undergraduate GPA, GRE verbal %, GRE quantitative %, GRE analytical %, Java, and Matlab. Conversely, applicant age and time since the last degree are two negative predictors. These findings are consistent with our observations during the MSDS and MSCS admissions process. That is, applicants with high undergraduate GPAs and GRE scores are likely to be admitted, while applicants who have been out of school for an extended period (i.e., with high age and time since degree values) are typically switching careers and lack the STEM background required by these programs, and thus are less likely to be admitted. However, it is notable and surprising that the GRE verbal score has higher importance than the quantitative score for STEM degree programs. One possible explanation is that there are many more very low GRE verbal scores than quantitative scores, and we are unlikely to admit students with extremely low scores of either type.

#### Using LOR data

Table 4 shows four features that made above-average contributions to the model’s outcomes when we used LORs to predict admissions decisions. The small number of principal predictors can be explained by two factors. First, the total number of features for the dataset is fewer than the structured data (i.e., 17 vs. 23). Second, the model’s performance is limited, implying weak predictive signals in the data and hence fewer strong predictors. Of these four features, three are generated by the NLU software (i.e., Sentiment, t-Sad, and t-Impolite), and the additional feature is the length of a letter. In terms of relative importance, a letter’s sentiment score is the highest-ranked feature, followed by the letter’s length and then the scores of sad and impolite tones.

**Fig 3:**
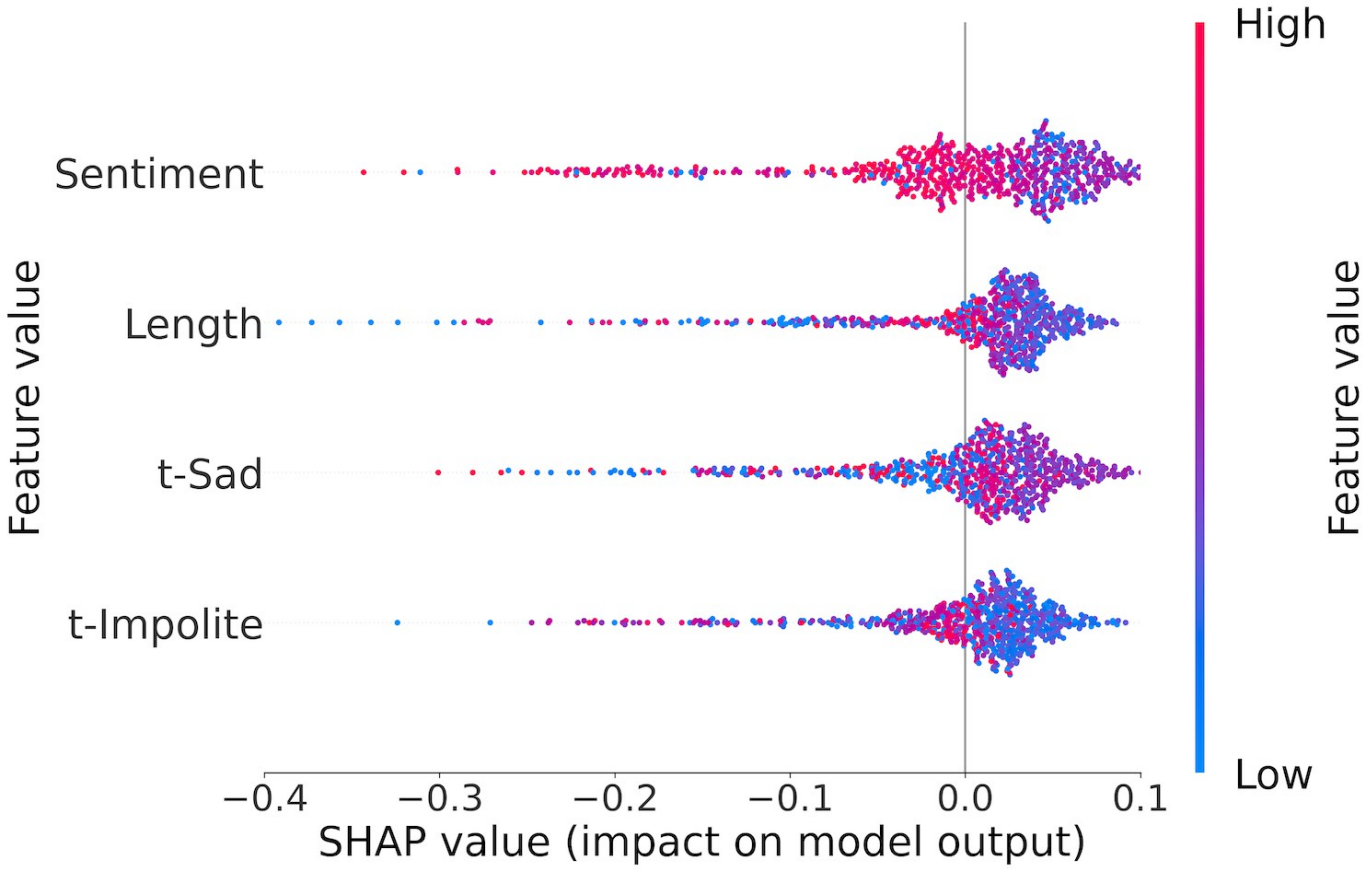
Principal predictors for admissions prediction using LOR data.

In studying the directional impact of these features in Fig 3, we observe that data values of the three NLU features form large blobs near the y-axis, suggesting weak directional impact signals. This finding is consistent with the model’s limited efficacy, which could yield counterintuitive or ambiguous directional contributions from the features. In particular, LORs with high sentiment scores are distributed more on the left side of the y-axis, indicating a negative impact on admissions decisions. The directional impact of a sad tone is unclear, and the impolite tone is seemingly a negative predictor. LOR length is the second highest ranked feature, and data points on the far left side of the y-axis (i.e., high negative contribution) consist of short LORs, indicating a negative influence on admissions decisions.

#### Using combined data

Table 4 lists the top ten of eighteen above-average predictive features for the combined model. Eight of them overlap with those principal predictors for the non-LOR model. The remaining two features, relevance and e-fear, are LOR features, and neither appears as an above-average feature when only the LORs are used. Given the importance of LOR relevance, it is reassuring that relevance is the top LOR feature and a positive predictor for the combined model. Furthermore, the top three predictors for the LORs model (i.e., sentiment, length, and sad tone) are also principal predictors for the combined model but ranked 11 to 13. While the order of these features may differ from that in those two models, the directional impacts remain the same.

**Fig 4:**
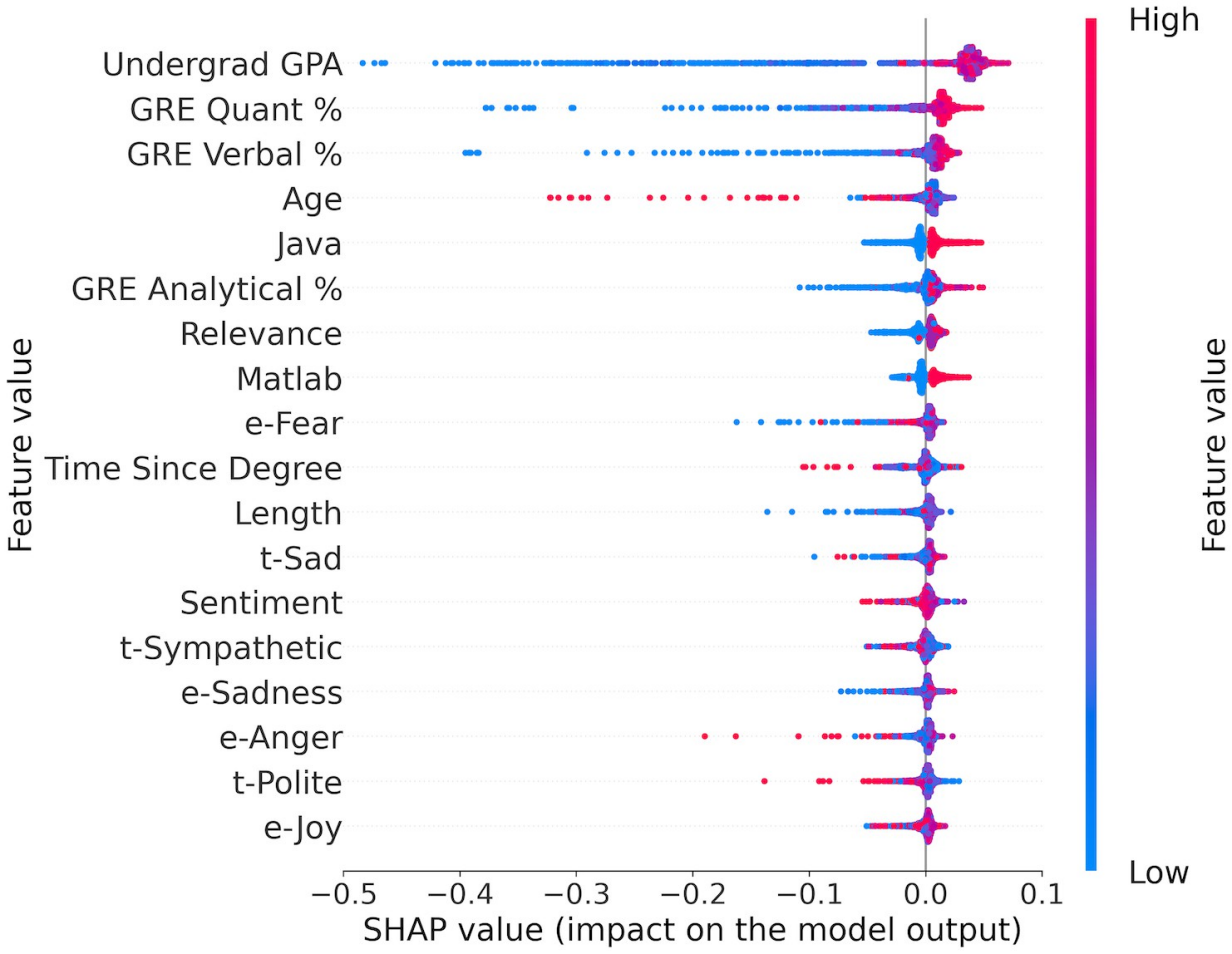
Principal predictors for admissions prediction using combined data.

The complete list of eighteen above-average predictors for the combined model, displayed in Fig 4, includes several LOR features generated by the NLU software. Four of these relate to emotions: fear, sadness, anger, and joy. Similar to other LOR features discussed in the previous subsection, these predictor values form large blobs near the y-axis, suggesting a less pronounced directional impact. Nevertheless, LORs with low fear scores constitute the left end of the x-axis, indicating a negative impact on admissions decisions, while the opposite trend is observed for anger. The model also utilizes scores for the sympathetic and polite tones, with a high score in the polite tone exhibiting a negative impact.

The fact that the above unique features for the combined model invariably originated from the LOR data confirms that the model’s superior performance relied on more effective usage of the LORs.

## Conclusion

In this study, we evaluated a machine learning approach toward making admissions decisions for two MS-level Computer and Information Science programs. We partitioned the application data into non-LOR and LORs components to better understand the impact of these data sources. Our approach exploits sophisticated NLP software and manual ratings to extract information from the LORs— which is a distinctive aspect of this research. Predictive models were generated using different sets of features to better understand the impact of various data sources on admissions decisions. The experiment results confirm the value of LORs for the models, and the SHAP analyses further validate the effectiveness of our LOR features.

This study provides promising results in utilizing automated systems for graduate admissions decisions. While we included manually rated features in our study, we implemented measures to control inter-rater variability, aiming to minimize human subjectivity. Additionally, ensemble learning algorithms, such as random forest, have the inherent ability to cancel random noises and enhance the correct decisions. Consequently, we can reasonably argue that the predictions made by our model are more consistent, and probably more objective, than decisions made by individual admission committee readers. Although the performance of our resulting model is not perfect, a predictive accuracy of 89% is sufficient to deploy the model as an auxiliary tool to assist admissions committee members. For example, the tool could be particularly useful for programs with a large numbers of applications, in which it could be used to filter out particularly weak applications, which could be discarded after a brief review. The tool could also be used to check the committee recommendations, so that when the tool issues an opposing recommendation, the committee members could take a second look to see if they might have missed something or been too hasty in their decision. The tool could also be used to break “ties” when admission committee members issue opposing recommendations; note that in such cases the tool would never yield a final decision that did not have human support. Alternatively, if some fully automated decisions are desired, the model’s high precision (97%) provides justification for automatically accepting the positive acceptance predictions.

Our study has several limitations. Firstly, the manual rating of some features of the LORs restricts the broader applicability of our models. One of our current projects explores automated methods to mitigate this limitation. Additionally, our study would have benefited from investigating the influence of factors such as the experience and prolificacy of the letter writers, as some previous research has shown their impact on the positivity of recommendations. Incorporating these factors into our analysis could provide a more comprehensive understanding of the dynamics involved in the LOR evaluation process. Furthermore, it is important to consider the intrinsic biases and personal relationships between application reviewers and letter writers, as these factors can introduce human subjectivity. Due to limitations in the NLP software, our analysis was restricted to generic features such as sentiment, tones, and emotions. Incorporating additional information, such as specific accomplishments or skills mentioned in the LORs, as we did for the resumes, could provide further insights and be a valuable future research endeavor. Despite its limitations, this study provides an initial step in developing a tool to assist with admissions decisions that incorporates textual data sources. We are highly motivated to continue this research and plan to extend our approach to include additional graduate programs, possibly some undergraduate programs, and examine the impact of LOR data on those admissions decisions.
